# Supplementary material for: O-GlcNAcylated p53 in the liver modulates hepatic glucose production
Source: Nat Commun. 2021 Aug 20;12:5068. doi: 10.1038/s41467-021-25390-0 (PMC8379189; doi:10.1038/s41467-021-25390-0)
Supplement: Supplementary file 1 — Supplementary Information [file 41467_2021_25390_MOESM1_ESM.pdf]

Supplementary figure 1.

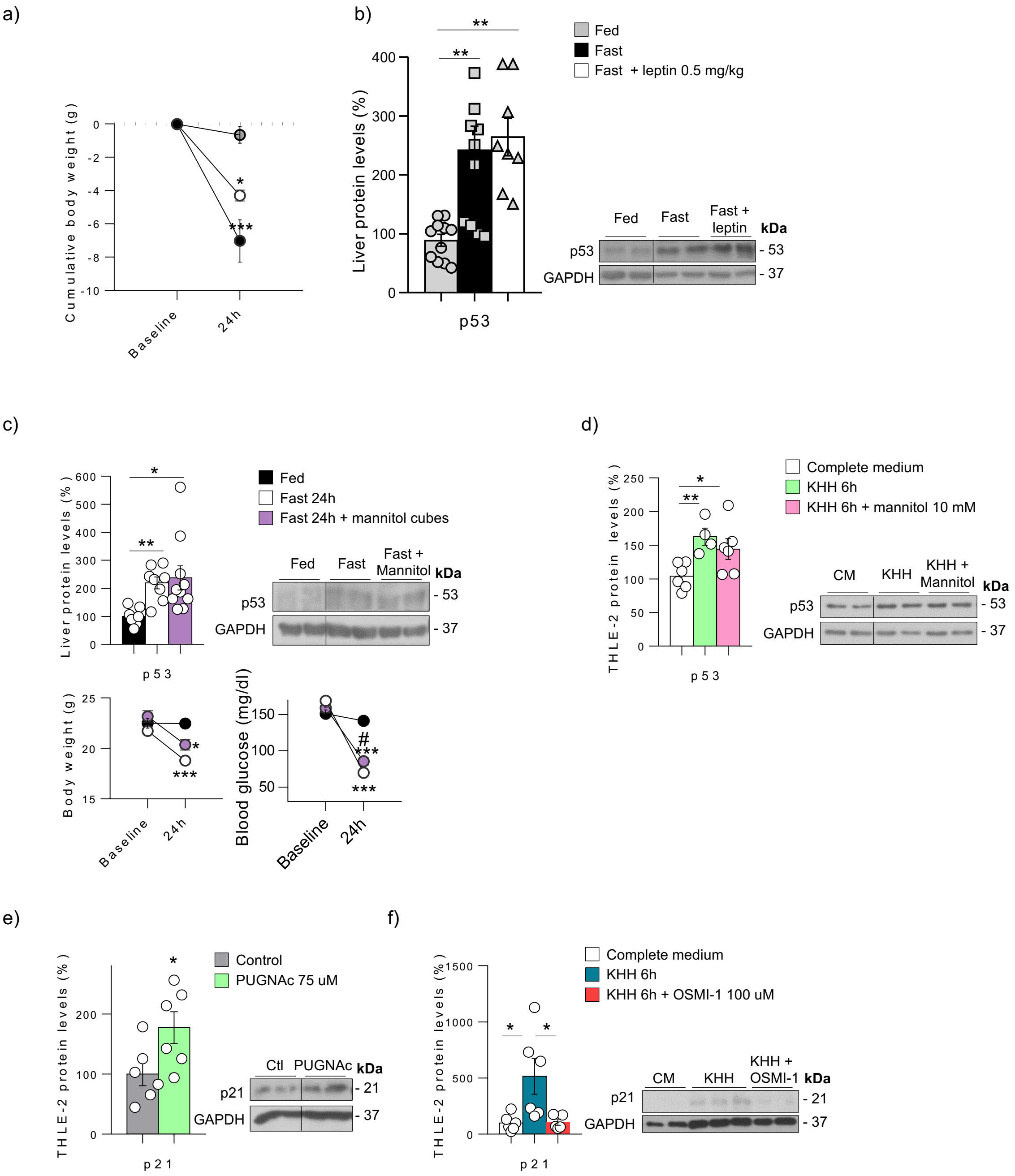

Supplementary figure 1. Hepatic p53 protein levels are not regulated by leptin and mannitol during fasting. WT animals were treated with saline or recombinant leptin (at a dose of 0.5 mg kg^{-1} of body weight every 12 h for 3 days (ip injection) (fed n = 12; fast n = 10; fast+leptin n = 8). **a)** Cumulative body weight change and **b)** hepatic p53 protein levels were assessed. **c)** p53 protein levels, body weight and blood glucose levels in mice *fed ad libitum*, fasted for 24 hours and fed with mannitol (fed n = 7; fast 24h n = 8; and fast 24h + mannitol n = 10). * depicts differences compared to fed group, and $^{\#}$ depicts differences between fast 24h and fast 24h + mannitol cubes groups. **d)** p53 protein levels in THLE-2 cells kept in complete medium (CM), KHH or KHH supplemented with mannitol 10 mM (CM n = 6; KHH 6h n = 4; and KHH 6h + mannitol n = 6). **e)** p21 protein levels in THLE-2 cells treated with PUGNAc 75 μM (n = 6). **f)** p21 protein levels in THLE-2 cells kept in complete medium or KHH, in the presence or absence of OSMI-1 100 μM (n = 6). Expression of GAPDH served as loading control, and control values were normalized to 100%. Dividing lines indicate splicings within the same gel. Data are presented as mean ± standard error mean (SEM). * and $^{\#}$ denotes P<0.05, ** denotes P<0.01, and *** denotes P<0.001, determined by two-tailed Student's t test (**e**) or one-way ANOVA followed by Bonferroni post-hoc testing (**a**, **b**, **c**, **d**, **e**, and **f**). "n" denotes independent animals or cell culture wells.

Supplementary figure 2.

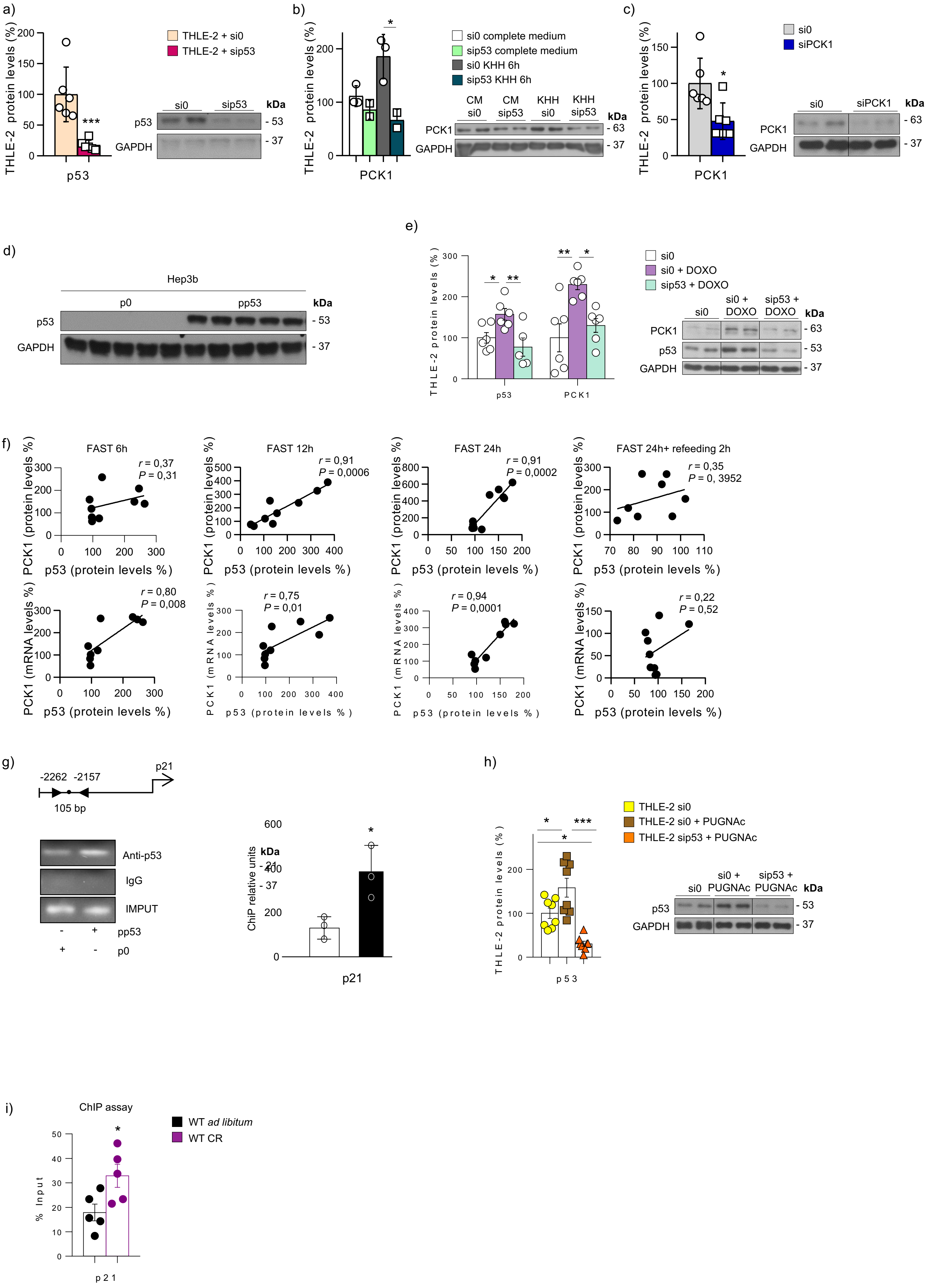

Supplementary figure 2. Transfection efficiency in THLE-2 and Hep3B cells. a) Protein levels of p53 in THLE-2 transfected with siRNA control or siRNA p53 (*n* = 6). **b)** PCK1 levels in THLE-2 cells transfected with siRNA control or siRNA p53 and then incubated with complete medium (CM) or KHH for 6 h (*n* = 3). **c)** PCK1 protein levels in THLE-2 transfected with siRNA control or siRNA PCK1 (*n* = 6). **d)** p53 levels in Hep3B cells transfected with empty plasmid or a plasmid encoding p53 (*n* = 6). **e)** p53 and PCK1 protein levels in THLE-2 transfected with siRNA control or siRNA p53 and then treated with doxorubicin 25 nM (*n* = 6). **f)** Correlations between p53 protein levels and PCK1 protein and mRNA levels during fasting. **g)** p21 promotor amplification and quantification after the ChIP assay performed in Hep3B cells (*n* = 3). **h)** p53 protein levels in THLE-2 cells transfected with siRNA control or siRNA p53 and then treated with PUGNAc 75 μM (*n* = 8). **i)** p21 promotor levels after a p53 ChIP assay in WT mice fed *ad libitum* or subjected to 60% calorie restriction (CR) (*n* = 5). Expression of GAPDH (western blot) or HPRT (qRT-PCR) served as loading control, and control values were normalized to 100%. Dividing lines indicate splicings within the same gel. Data are presented as mean ± standard error mean (SEM). * denotes $P<0.05$, ** denotes $P<0.01$, and *** denotes $P<0.001$, determined by two-tailed Student's t test (**a**, **c**, **g**, and **i**) or one-way ANOVA followed by Bonferroni post-hoc testing (**b**, **e**, and **h**). "*n*" denotes independent animals or cell culture wells.

Supplementary figure 3.

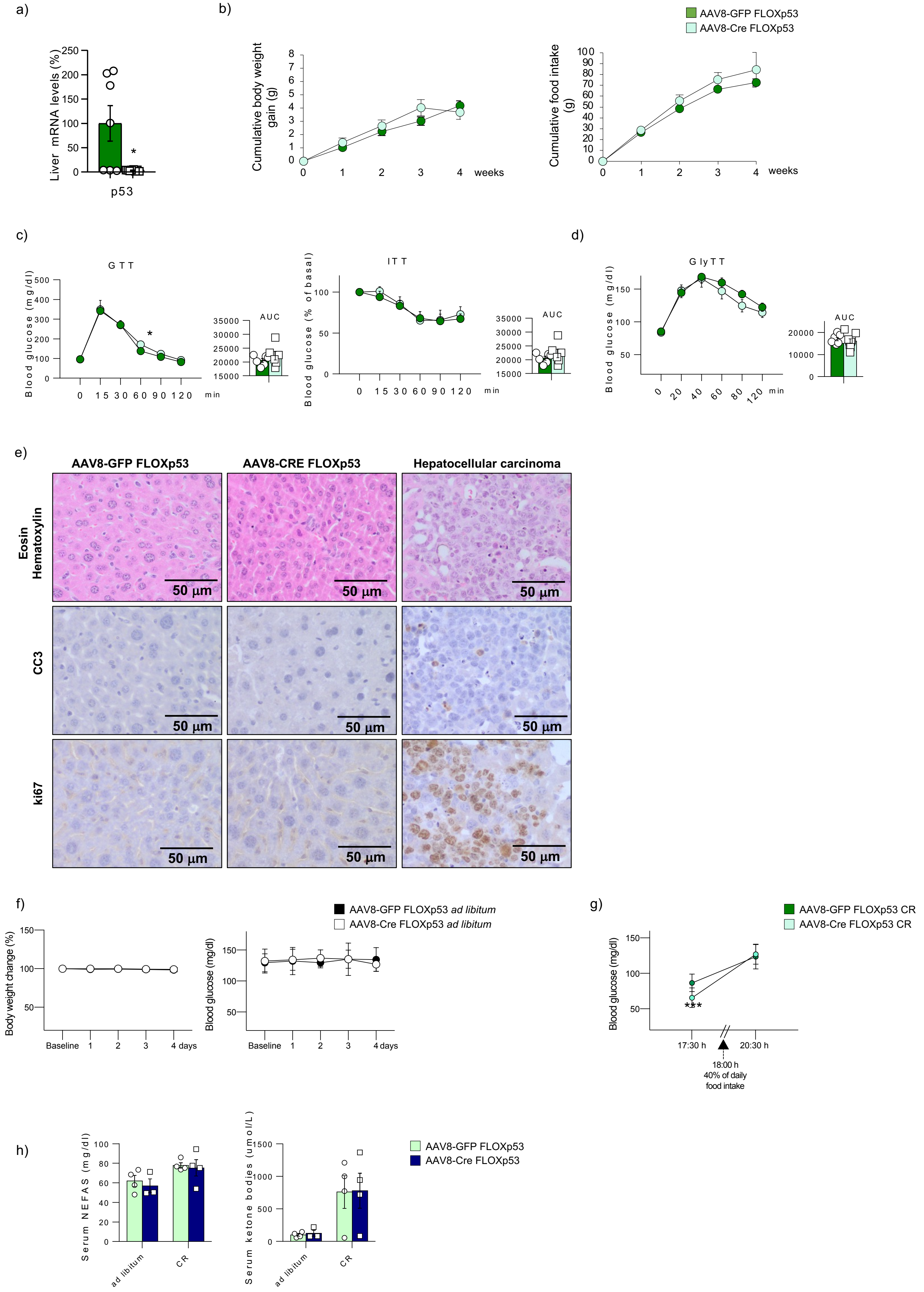

Supplementary figure 3. AAV8-GFP and AAV8-Cre FLOXp53 mice. p53floxed mice were injected into the tail vein with either AAV8-GFP (control group) or AAV8-Cre (n = 8). **a)** *p53* mRNA levels in the liver. **b)** Body weight change and food intake four weeks after tail vein injection. **c)** Glucose tolerance test (GTT) and insulin tolerance test (ITT). **d)** Glycerol tolerance test (GlyTT). **e)** Hematoxylin and Eosin (upper panels), cleaved caspase 3 (CC3) (central panels) and ki67 (lower panels) staining of liver sections. Images are representative of two independent experiments. **f)** Body weight change and food intake in mice fed *ad libitum* **g)** Blood glucose after the daily meal during calorie restriction (CR). **h)** Serum NEFAs and ketone bodies in mice fed *ad libitum* and mice under calorie restriction (n = 4). Area under the curve (AUC) is provided. HPRT (qRT-PCR) served as loading control, and control values were normalized to 100%. Data are presented as mean ± standard error mean (SEM). * denotes P<0.05 and *** denotes P<0.001, determined by two-tailed Student's t test. "n" denotes independent animals.

Supplementary figure 4.

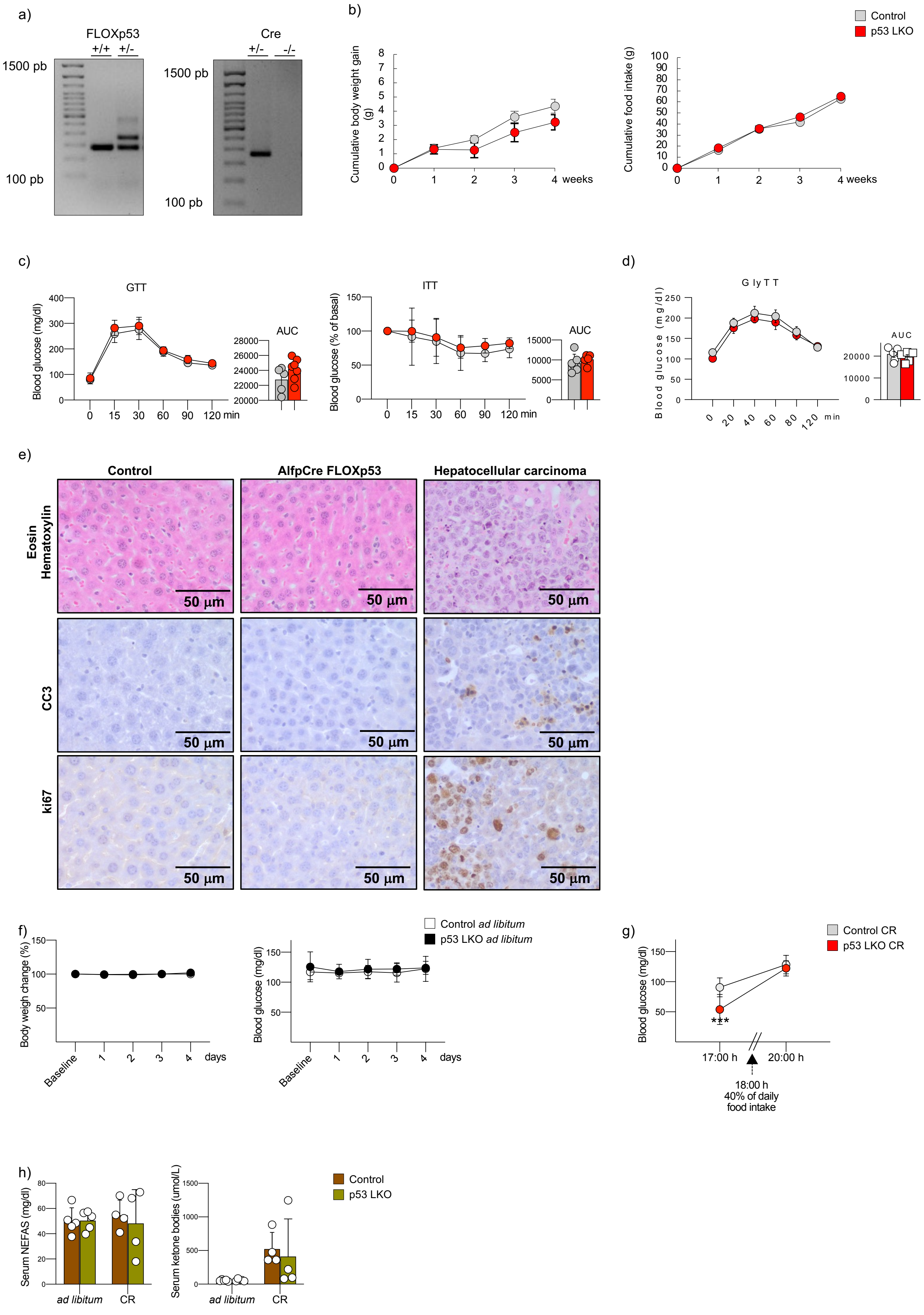

Supplementary figure 4. p53 LKO mice. p53 LKO mice and their control littermates (control *n* = 5; LKO *n* = 7)**. a)** Genotyping of p53 LKO mice (FLOXp53 +/+ and Cre +/-) and their control littermates. Images are representative of two independent genotype determinations. **b)** Body weight change and food intake before the beginning of the experiments. **c)** Glucose tolerance test (GTT) and insulin tolerance test (ITT). d) Glycerol tolerance test (GlyTT). **e)** Hematoxylin and Eosin (upper panels), CC3 (central panels) and ki67 (lower panels) stainings of liver sections. Images are representative of two independent experiments. **f)** Body weight change and food intake in mice fed *ad libitum*. **g)** Blood glucose after the daily meal during calorie restriction (CR). **h)** Serum NEFAs and ketone bodies in mice fed ad libitum and mice under calorie restriction (*n* = 5). Area under the curve (AUC) is provided. Data are presented as mean ± standard error mean (SEM). *** denotes *P*<0.001, determined by two-tailed Student's t test. "*n*" denotes independent animals.

Supplementary figure 5.

a)

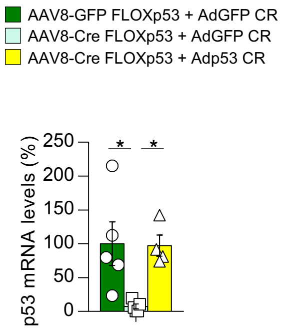

b)

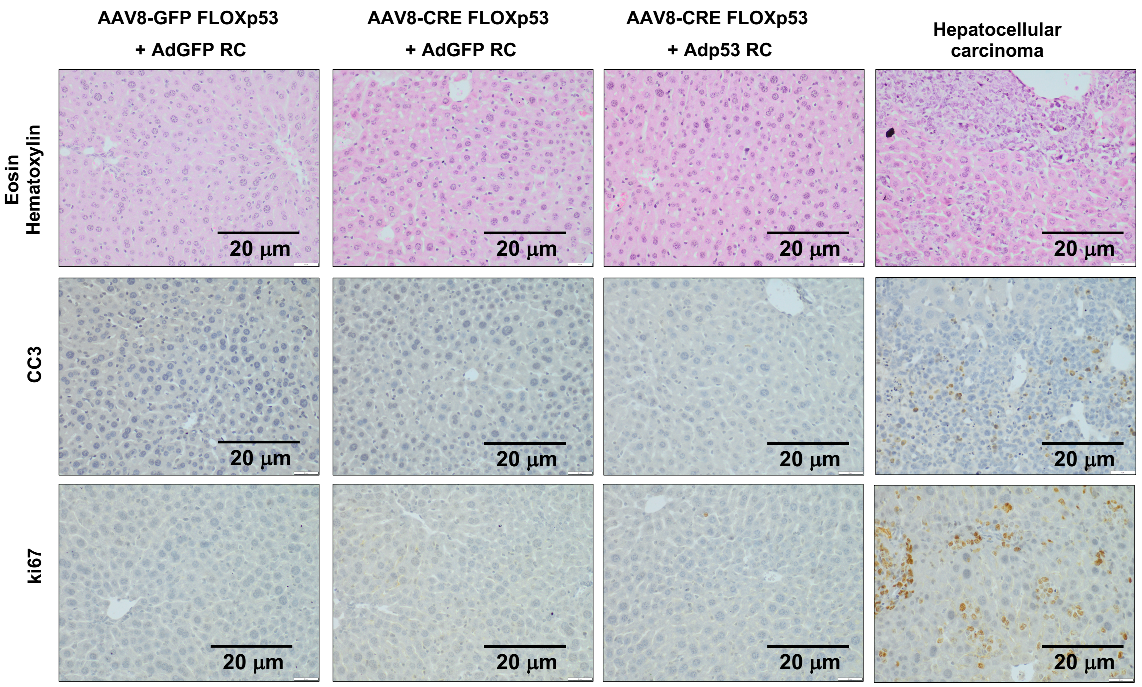

c)

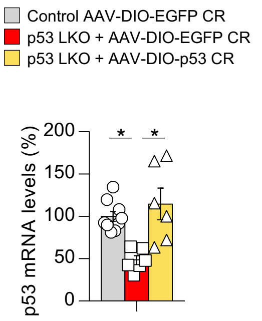

d)

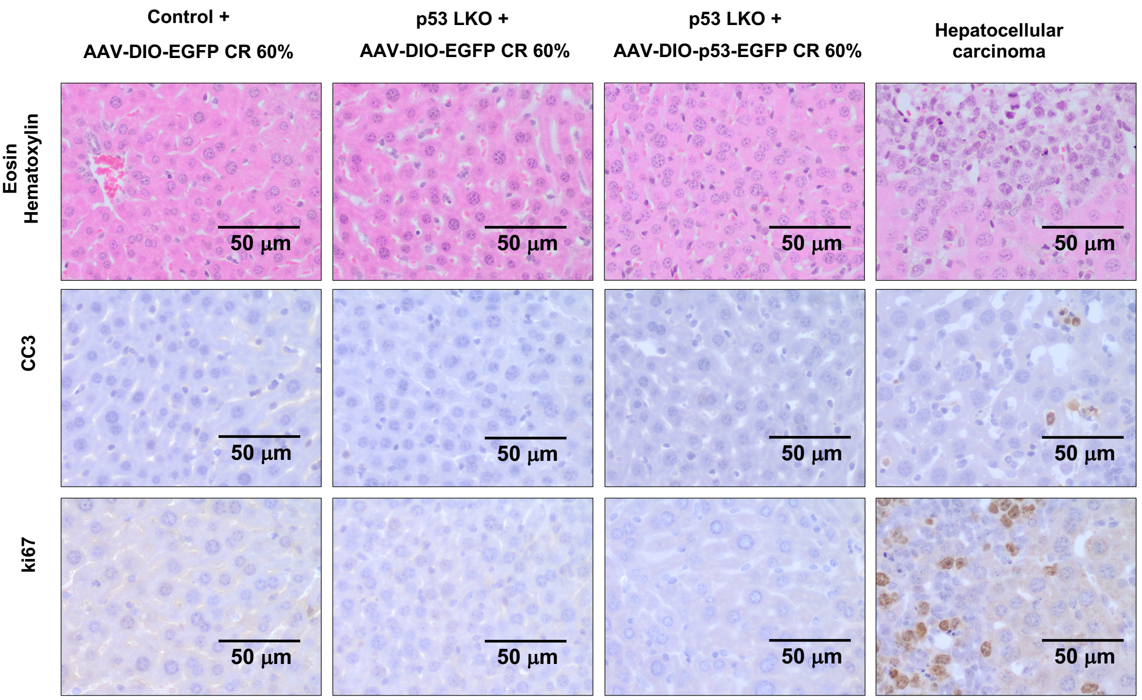

e)

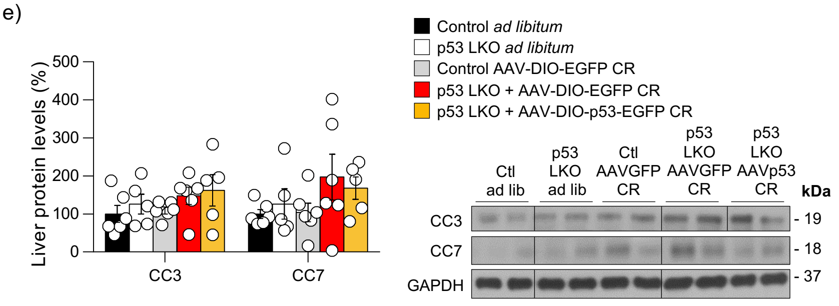

Supplementary figure 5. p53 rescue in hepatic p53 null mice models does not affect proliferation or apoptosis. *p53* mRNA levels in **a)** p53floxed mice injected firstly with AAV8-GFP or AAV8-Cre, and then with Adp53 to rescue the expression of p53 in the liver of hepatic p53 deficient mice (AAV8-GFP + AdGFP n = 5; AAV8-Cre + AdGFP n = 7; and AAV8-Cre + Adp53 n = 5); and in **c)** control and p53 LKO mice injected with AAV-DIO-EGFP or AAV-DIO-p53 to rescue the expression of p53 in their hepatocytes (control + AAV-EGFP n = 9; LKO + AAV-EGFP n = 7; and LKO + AAV-p53 n=6). Hematoxylin and Eosin (upper panels), CC3 (central panels) and ki67 (lower panels) stainings of liver sections in **b)** p53floxed mice injected into the tail vein with either AAV8-GFP (control group) or AAV8-Cre, and then injected with an adenovirus encoding GFP (AdGFP) or p53 (Adp53), and **d)** control mice and p53 LKO mice injected with an AAV-DIO-EGFP or AAV-DIO-p53-EGFP. Images are representative of two independent experiments. **e)** CC3 and CC7 protein levels in the liver of mice explained above (n = 6). GAPDH (western blot) or HPRT (qRT-PCR) served as loading control, and control values were normalized to 100%. Dividing lines indicate splicings within the same gel. Data are presented as mean ± standard error mean (SEM). * denotes P<0.001, determined by one-way ANOVA followed by Bonferroni post-hoc testing. "n" denotes independent animals.

Supplementary figure 6.

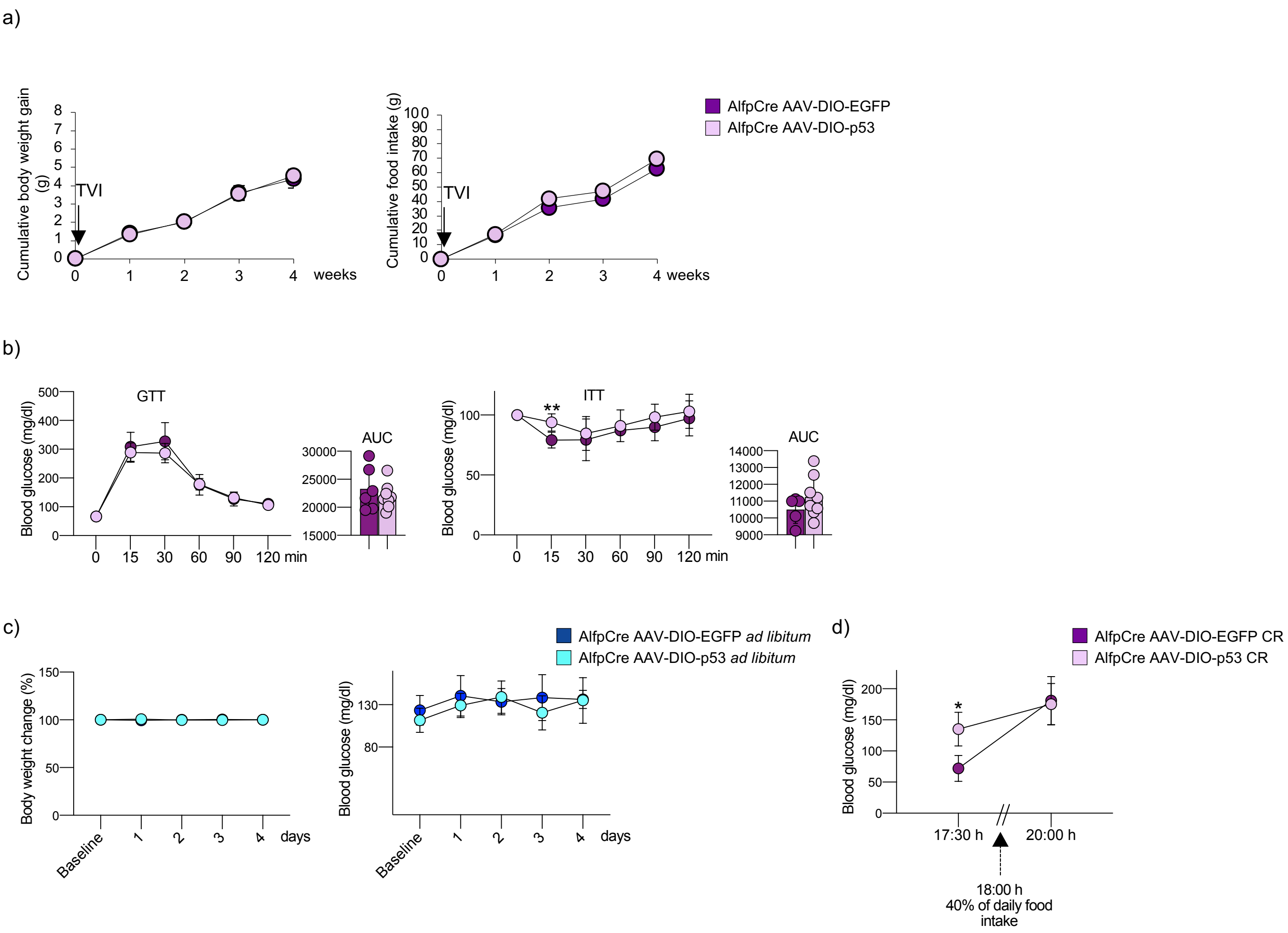

Supplementary figure 6. AAV- DIO- GFP and AAV-DIO-p53 AlfpCre mice. AlfpCre mice were injected into the tail vein with either AAV-DIO-EGFP (control group) or AAV-DIO-p53 (AAV-EGFP *n* = 7; and AAV-p53 *n* = 8 independent animals per group). **a)** Body weight change and food intake four weeks after tail vein injection. **b)** Glucose tolerance test (GTT) and insulin tolerance test (ITT). **c)** Body weight change and food intake in mice fed *ad libitum*. **d)** Blood glucose after the daily meal during calorie restriction (CR). Area under the curve (AUC) is provided. Data are presented as mean ± standard error mean (SEM). * denotes $P<0.05$ and ** denotes $P<0.01$, determined by two-tailed Student's t test (**b** and **d**).

Supplementary figure 7 .

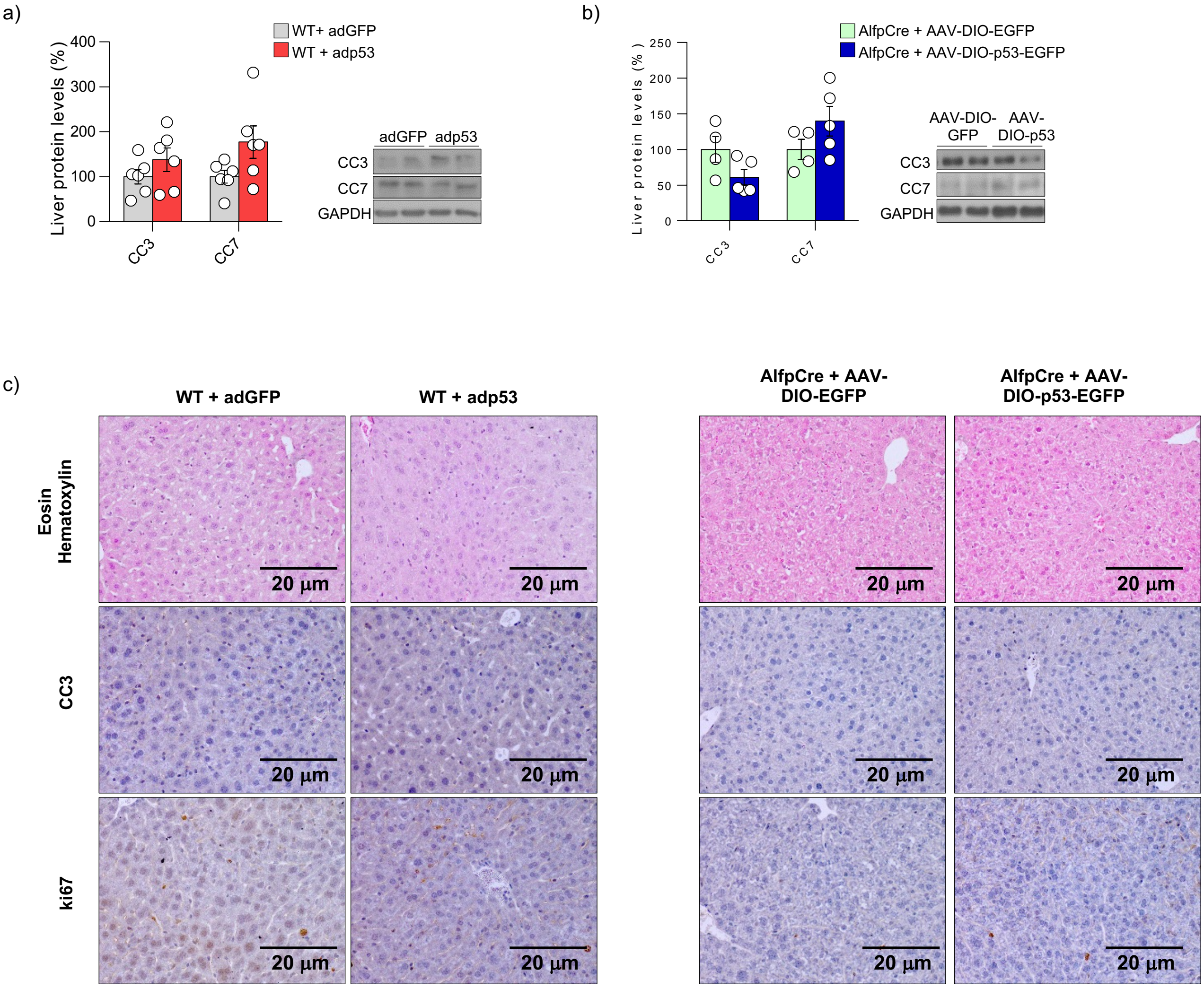

Supplementary figure 7: p53 overexpression in WT and AlfpCre mice does not alter apoptosis or proliferation. CC3 and CC7 protein levels in **a)** WT mice injected with adGFP or adp53 (n=6 per group), and **b)** AlfpCre mice injected with AAV-DIO-EGFP or AAV-DIO-p53 (AAV-GFP n = 4; and AAV-p53 n = 5 independent animals per group). **c)** Hematoxylin and Eosin (upper panels), CC3 (central panels) and ki67 (lower panels) staining of liver sections in WT mice injected with adGFP or adp53, and AlfpCre mice injected with AAV-DIO-EGFP or AAV-DIO-p53. Images are representative of two independent experiments. GAPDH served as loading control, and control values were normalized to 100%. Dividing lines indicate splicings within the same gel. Data are presented as mean ± standard error mean (SEM). "*n*" denotes independent animals

Supplementary figure 8 .

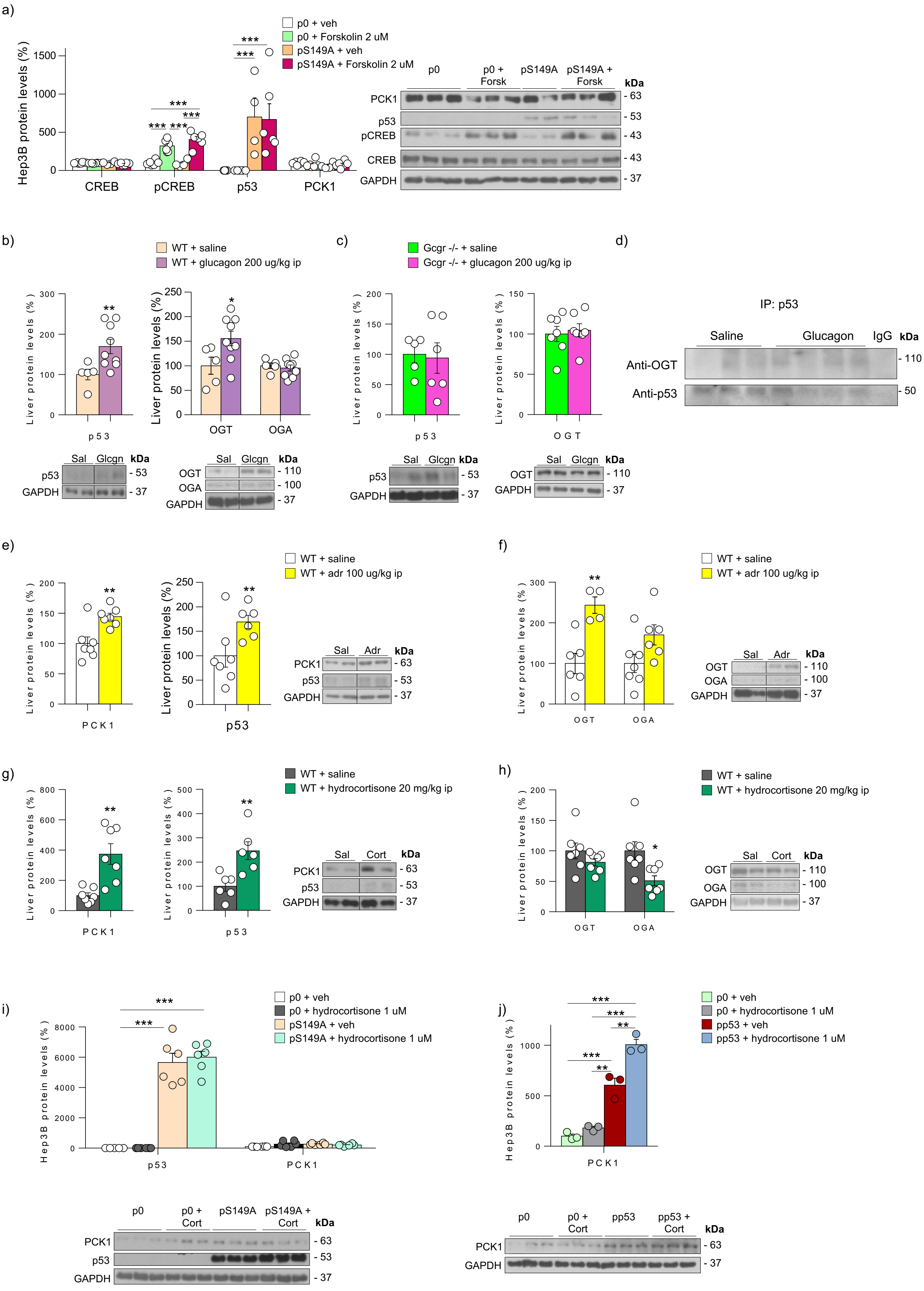

Supplementary figure 8. *O*-GlcNAcylation and p53 are regulated by glucagon, adrenaline and cortisol. a) CREB, pCREB, p53 and PCK1 protein levels in Hep3B cells transfected with p0 or a plasmid encoding p53-S149A, and then treated with vehicle or forskolin 2 µM (p0 + veh *n* = 6; p0 + forskolin *n* = 6; pS149A + veh *n* = 4; pS149A + forskolin *n* = 6). p53, OGT and OGA protein levels in **b)** WT mice treated with saline or glucagon (200 µg kg^{-1}) (WT + saline *n* = 5; and WT + glucagon *n* = 9) and **c)** in glucagon receptor KO mice (Gcgr -/-) treated with saline or glucagon (200 µg kg^{-1}) ip (Gcgr -/- *n* = 5; and Gcgr -/- + glucagon *n* = 6). **d)** p53 and OGT protein levels after p53 immunoprecipitation (saline *n* = 3; and glucagon *n* = 4). Image is representative of two independent experiments. PCK1, p53, OGT and OGA protein levels in WT mice treated with **e-f)** saline or adrenaline (100 µg kg^{-1}) (*n* = 7) and **g-h)** saline or hydrocortisone 20 mg kg^{-1} (*n* = 7). **i)** p53 and PCK1 protein levels in Hep3B cells transfected with p0 or a plasmid encoding p53-S149A, and then treated with PBS or hydrocortisone 1 µM (*n* = 6). **j)** PCK1 protein levels in Hep3B cells transfected wit p0 or a plasmid encoding WT p53 (pp53), and then treated with hydrocortisone 1 µM (*n* = 6). GAPDH served as loading control, and control values were normalized to 100%. Dividing lines indicate splicings within the same gel. Data are presented as mean ± standard error mean (SEM). * denotes $P<0.05$, ** denotes $P<0.01$, and *** denotes $P<0.001$, determined by two-tailed Student's t test (**b**, **e**, **f**, **g** and **h**) or one-way ANOVA followed by Bonferroni post-hoc testing (**a**, **i**, and **j**). "*n*" denotes independent animals or cell culture wells.

Supplementary figure 9.

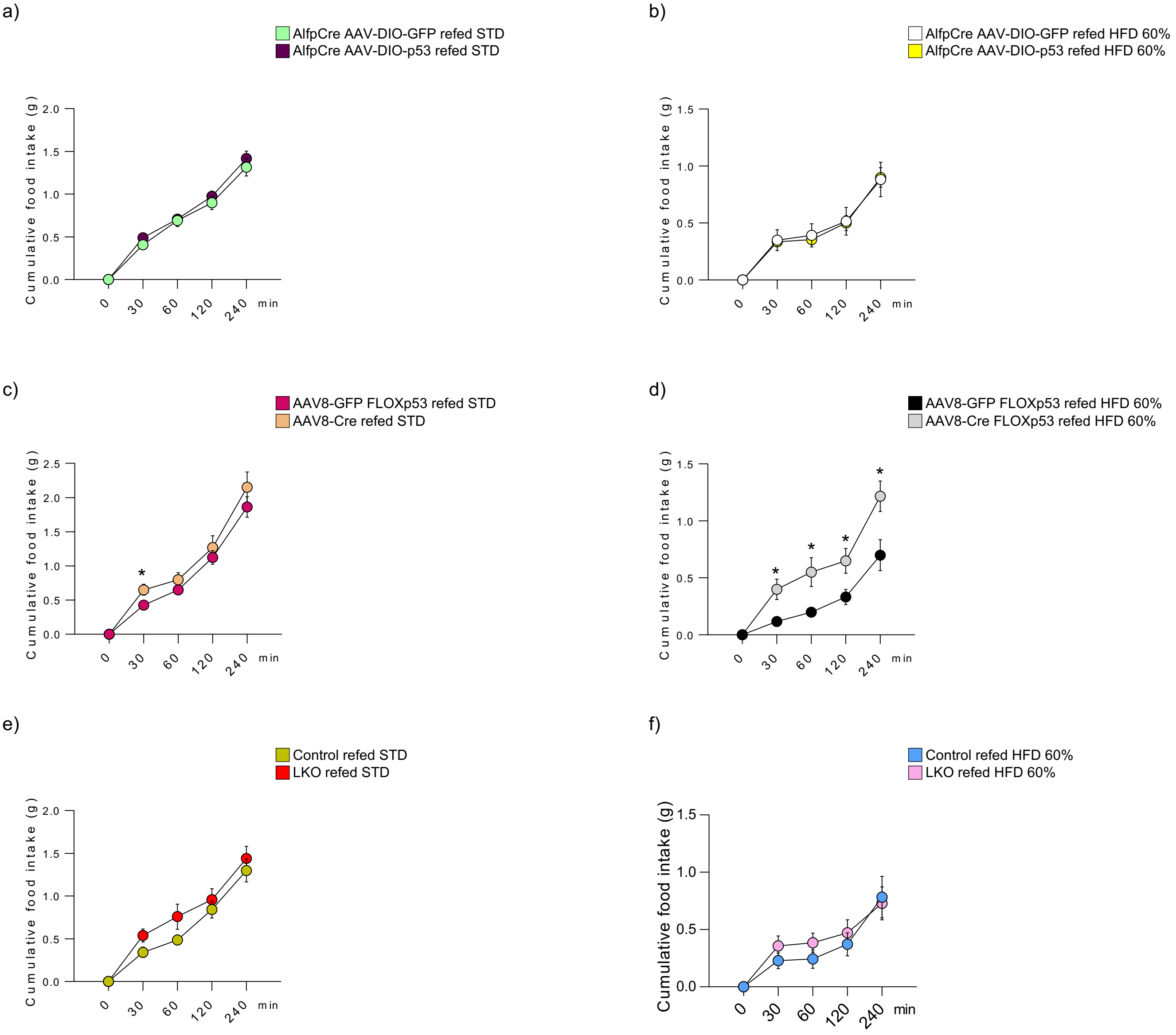

Supplementary figure 9. Food intake during refeeding with chow diet and HFD. AlfpCre +/- mice injected with AAV-DIO expressing either GFP or p53 refed with **a)** chow diet (*n* = 12) and **b)** HFD after an overnight fasting (*n* = 12). FLOXp53 mice injected with AAV8 expressing either GFP or Cre refed with **c)** chow diet (*n* = 8) and **d)** HFD (*n* = 8) after an overnight fasting. p53 LKO mice refed with **e)** chow (control *n* = 8; and LKO *n* = 6) diet and **f)** HFD (control *n* = 8; and LKO *n* = 8) after an overnight fasting. Data are presented as mean ± standard error mean (SEM). * denotes $P<0.05$ determined by two-tailed Student's t test. "*n*" denotes independent animals.

Supplementary figure 10.

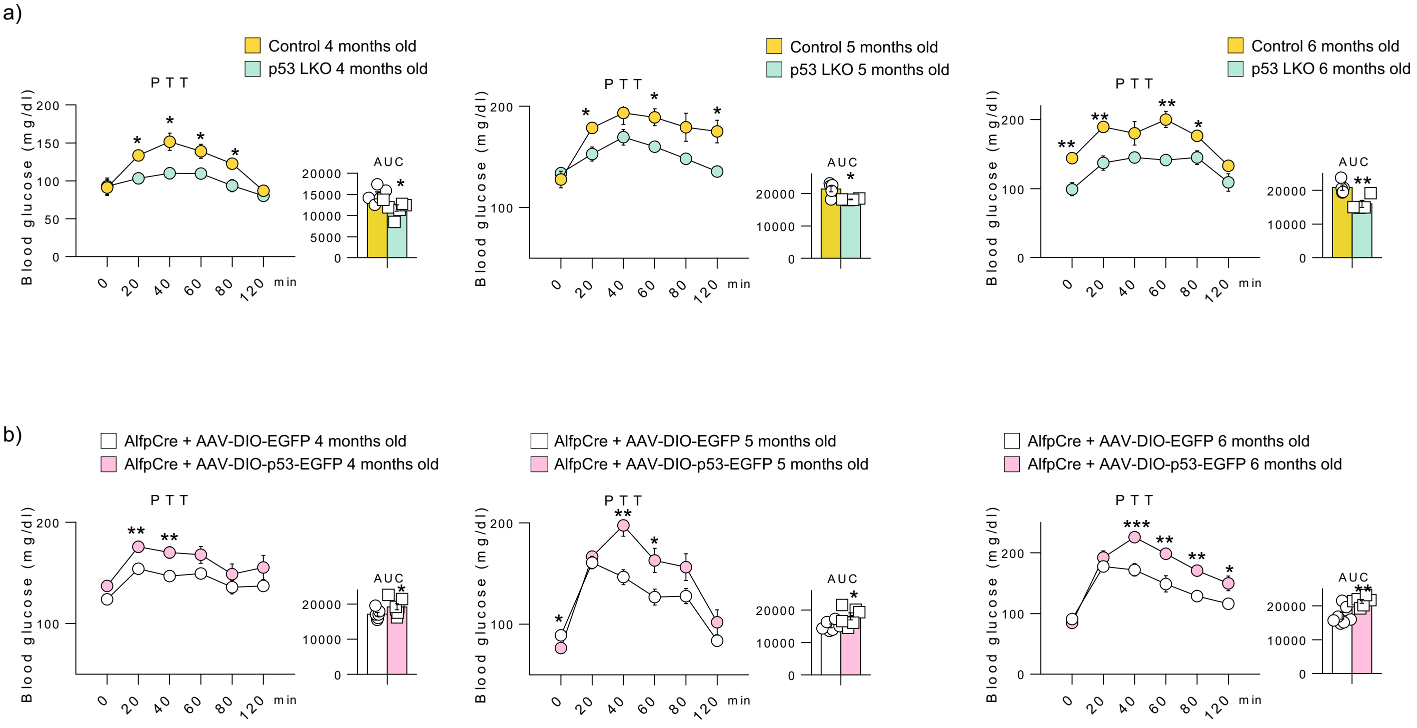

Supplementary figure 10. Hepatic p53 manipulation affects glucose production at long term. a) Blood glucose levels after PTT in 4-, 5- and 6-months old control and LKO mice (control *n* = 5, and LKO *n* = 6 independent animals per group). **b)** Blood glucose levels after PTT in 4, 5 and 6 months old AlfpCre mice, injected with AAV-DIO-EGFP or AAV-DIO-p53-EGFP (n = 7 independent animals per group). Area under the curve (AUC) is provided. Data are presented as mean ± standard error mean (SEM). * denotes $P<0.05$, ** denotes $P<0.01$, and *** denotes $P<0.001$, determined by two-tailed Student's t test. "*n*" denotes independent animals.

Supplementary Table 1. Univariate analysis of the correlations between hepatic p53 and PCK1 transcript and protein levels and variables related to glucose metabolism.

	P53 mRNA		P53 protein		*PCK1* mRNA		PCK1 protein	
	r	*P*	*r*	*P*	*r*	*P*	*r*	*P*
Glucose	-0.24	0.109	0.22	0.156	-0.18	0.214	0.04	0.826
Glucose 2 h OGTT	-0.02	0.921	0.59	**0.002**	0.12	0.571	0.32	0.137
Insulin	-0.03	0.825	0.29	0.054	-0.08	0.600	0.06	0.712
Insulin 2 h OGTT	0.16	0.461	-0.09	0.680	0.48	**0.012**	-0.08	0.708
HOMA	-0.08	0.623	0.32	**0.030**	-0.14	0.355	0.09	0.585
QUICKI	0.26	0.085	-0.10	0.531	-0.14	0.361	0.02	0.887

Values are Pearson's correlation coefficients and associated *P* values calculated by a two-tailed test after age-, sex- and BMI-adjustment. OGTT, oral glucose tolerance test; HOMA, homeostasis model assessment; QUICKI, quantitative insulin sensitivity check index.

Supplementary Table 2. Multiple linear regression analyses with hepatic p53 and PCK1 mRNA and protein as dependent variables for all subjects in the cross-sectional study.

	p53 mRNA		p53 protein		*PCK1* mRNA		PCK1 protein	
Model I	*β*	*P*	*β*	*P*	*β*	*P*	*β*	*P*
Age	1.009	0.554	2.976	**0.004**	-1.522	0.181	1.288	0.179
Sex	-34.226	0.374	21.819	0.324	-20.473	0.415	11.256	0.612
BMI	-0.455	0.872	1.901	0.206	-1.340	0.440	1.056	0.502
HOMA	-0.224	0.863	1.582	**0.037**	-0.756	0.387	0.181	0.950
AST	-2.519	0.662	-4.995	0.137	1.826	0.614	-5.406	0.139
ALT	1.737	0.624	2.701	0.187	-1.798	0.427	3.351	0.103
Adjusted R^2	0.049	0.921	0.218	**0.017**	-0.040	0.654	-0.021	0.529
Model II	*β*	*P*	*β*	*P*	*β*	*P*	*β*	*P*
Age	0.921	0.588	3.601	**0.004**	-1.600	0.145	1.112	0.221
Sex	-45.345	0.223	14.281	0.517	-11.234	0.631	15.308	0.450
BMI	-0.472	0.867	2.420	0.122	-1.372	0.416	1.255	0.418
AST	-1.394	0.808	-4.105	0.233	2.013	0.565	-5.272	0.107
ALT	1.310	0.706	1.716	0.404	-2.100	0.329	3.210	0.083
Adjusted R^2	0.064	0.719	0.140	**0.046**	-0.018	0.538	0.003	0.419
Model III	*β*	*P*	*β*	*P*	*β*	*P*	*β*	*P*
Age	0.679	0.651	2.462	**0.008**	-1.372	0.191	1.243	0.188
Sex	-31.412	0.337	5.904	0.762	-11.978	0.594	17.798	0.431
BMI	-1.085	0.669	2.041	0.163	-1.908	0.246	1.908	0.240
HOMA	-0.292	0.812	1.577	**0.034**	-0.788	0.358	1.398	0.587
Adjusted R^2	0.028	0.863	0.208	**0.008**	-0.005	0.314	-0.014	0.450

BMI, body mass index; HOMA, homeostasis model assessment. *β* is the regression coefficient, which allows evaluating the relative significance of each independent variable in multiple linear regression analyses. Adjusted R^2 expresses the percentage of the variance explained by the independent variables in the different models (i.e. 0.208 is 20.8%). Statistical significant values are in bold.

Supplementary Table 3. Univariate analysis of the correlations between mRNA expression of genes involved in hepatic *O*-GlcNAcylation and variables related to glucose metabolism.

	OGT mRNA		*OGA* mRNA		*GFAT1* mRNA		*GFAT2* mRNA	
	r	*P*	*r*	*P*	*r*	*P*	*r*	*P*
Glucose	0.28	0.070	0.01	0.922	0.31	**0.049**	0.24	0.118
Glucose 2 h OGTT	0.26	0.223	0.34	0.102	0.50	**0.018**	0.49	**0.012**
Insulin	0.07	0.669	0.02	0.888	0.09	0.592	0.35	**0.021**
Insulin 2 h OGTT	0.16	0.459	0.29	0.177	0.10	0.653	0.28	0.170
HOMA	0.11	0.471	0.07	0.962	0.14	0.377	0.39	**0.009**
QUICKI	-0.25	0.104	-0.06	0.691	-0.22	0.172	-0.41	**0.006**

OGT, *O*-linked N-acetylglucosamine (GlcNAc) transferase; *OGA*, OGA *O*-GlcNAcase; *GFAT*, glutamine-fructose-6-phosphate transaminase. Values are Pearson's correlation coefficients and associated *P* values calculated by a two-tailed test after age-, sex- and BMI-adjustment. OGTT, oral glucose tolerance test; HOMA, homeostasis model assessment; QUICKI, quantitative insulin sensitivity check index.

Supplementary Table 4. Multiple linear regression analyses with gene expression of factors involved in hepatic O-GlcNAcylation as dependent variables for all subjects in the cross-sectional study.

	OGT mRNA		*OGA* mRNA		*GFAT1* mRNA		*GFAT2* mRNA	
Model I	*β*	*P*	*β*	*P*	*β*	*P*	*β*	*P*
Age	-2.115	0.123	-2.398	0.067	0.667	0.821	0.615	0.841
Sex	35.188	0.248	6.219	0.825	59.276	0.335	90.926	0.146
BMI	2.344	0.261	0.104	0.955	2.227	0.598	0.729	0.871
HOMA	0.933	0.363	0.120	0.901	1.632	0.404	5.838	**0.007**
AST	0.274	0.948	2.966	0.466	1.569	0.847	13.084	0.140
ALT	-0.332	0.901	-1.243	0.621	-0.566	0.912	-3.782	0.490
Adjusted R^2	0.149	0.391	0.097	0.681	0.055	0.915	0.279	**0.048**
Model II	*β*	*P*	*β*	*P*	*β*	*P*	*β*	*P*
Age	-1.885	0.151	-2.333	0.069	0.568	0.839	0.797	0.803
Sex	25.412	0.371	1.546	0.955	52.876	0.344	66.616	0.285
BMI	2.563	0.203	0.266	0.895	2.789	0.488	2.150	0.646
AST	0.928	0.819	4.259	0.289	2.130	0.782	12.899	0.160
ALT	-0.883	0.726	-2.315	0.344	-1.170	0.807	-4.350	0.438
Adjusted R^2	0.117	0.384	0.094	0.539	0.041	0.894	0.124	0.344
Model III	*β*	*P*	*β*	*P*	*β*	*P*	*β*	*P*
Age	0.015	0.994	-1.899	0.141	0.930	0.732	1.730	0.557
Sex	78.799	0.090	6.899	0.803	69.053	0.203	117.849	**0.045**
BMI	3.909	0.232	1.030	0.607	2.314	0.547	0.866	0.839
HOMA	1.058	0.526	-0.008	0.993	1.493	0.422	5.591	**0.010**
Adjusted R^2	0.086	0.422	0.057	0.639	0.057	0.680	0.198	0.055

BMI, body mass index; HOMA, homeostasis model assessment. β is the regression coefficient, which allows evaluating the relative significance of each independent variable in multiple linear regression analyses. Adjusted R^2 expresses the percentage of the variance explained by the independent variables in the different models (i.e. 0.208 is 20.8%). Statistical significant values are in bold.

Supplementary Table 5. The NAFLD activity score (NAS).

Feature	Description	Score
Steatosis (%)	<5	0
	5-33	1
	33-66	2
	>66	3
Lobular inflammation	No	0
	≤2 foci	1
	2-4 foci	2
	≥4 foci	3
Hepatocyte ballooning	No	0
	Moderate ballooning	1
	Evident ballooning	2

NAS ranges from 0 to 8 and is the sum of the separate scores for steatosis (0–3), lobular inflammation (0–3) and hepatocellular ballooning (0–2).

Supplementary Table 6. Univariate analysis of the correlations between hepatic p53 and PCK1 transcript and protein levels and NAFLD activity score (NAS).

	P53 mRNA		P53 protein		*PCK1* mRNA		PCK1 protein	
	r	*P*	*r*	*P*	*r*	*P*	*r*	*P*
NAS score	0.14	0.406	0.10	0.556	-0.18	0.286	-0.10	0.584

Values are Pearson's correlation coefficients and associated *P* values calculated by a two-tailed test after age-, sex- and BMI-adjustment.

Supplementary Table 7. Primers used for gene amplification.

Name	Primer sequence 5’ -> 3’
Human p53	FW: GTTCCGAGAGCTGAATGAGG<br>RV: TCTGAGTCAGGCCCTTCTGT
Human PCK1	FW: CATGATGGAGCTTTTCAGCA<br>RV: GAACACTTGCCCTCTCTTGC
Human OGT	FW: CATCGAGAATATCAGGCAGGAG<br>RV: CCTTCGACACTGGAAGTGTATAG
Human MGEA 5 (OGA)	FW: TTCACTGAAGGCTAATGGCTCCCG<br>RV: ATGTCACAGGCTCCGACCAAGT
Human GFAT1 (GFPT1)	FW: CGGGAAAGTCAAGATACCAGC<br>RV: CGTACACCAATCAACAGAGGG
Human GFAT2 (GFPT2)	FW: GATACAGAGACCATCGCCAAG<br>RV: GACACTCTTGAAAACCAGCG
Human HPRT	FW: ACCCCACGAAGTGTTGGATA<br>RV: AAGCAGATGGCCACAGAACT
Mus musculus p53	FW: CCTGTGCAGTTGTGGGTCAG<br>RV: GCTCTCTTTGCGCTCCCTG
Mus musculus PCK1	FW: CACCATCACCTCCTGGAAGA<br>RV: GGGTGCAGAATCTCGAGTTG
Mus musculus HPRT	FW: AAGCTTGCTGGTGAAAAGGA<br>RV: TTGCGCTCATCTTAGGCTTT

Supplementary Table 8. Antibodies used for western blot.

Protein target	Manufacter (catalog number)	Species reactivity	Dilution
p53	Cell Signaling (2524)	Mouse monoclonal	1:1000
Phosphoenolpyruvate Carboxykinase 1 (PCK1)	Abcam (ab70358)	Rabbit polyclonal	1:1000
O-linked N-Acetylglucosamine (O-GlucNAc)	Abcam (ab2739)	Mouse monoclonal	1:1000
Glyceraldehyde 3-phosphate Dehydrogenase (GAPDH)	Merck (CB1001)	Mouse monoclonal	1:5000
Phospho-CREB (pCREB) (Ser 133)	Cell Signaling (9198)	Rabbit monoclonal	1:1000
Phospho-AKT (pAKT) (Ser 473)	Cell Signaling (9271)	Rabbit polyclonal	1:1000
Phospho-Pyruvate Dehydrogenase E1-alpha subunit antibody (pPDH)	Abcam (ab177461)	Rabbit monoclonal	1:1000
Pyruvate Carboxylase (PC)	Abcam (ab128952)	Rabbit monoclonal	1:1000
Glucose-6-phosphatase	Abcam (ab83690)	Rabbit polyclonal	1:1000
p21	Cell Signaling (2947T)	Rabbit monoclonal	1:1000
O-GlcNAc transferase (OGT)	Cell Signaling (D1D8Q)	Rabbit monoclonal	1:1000

Supplementary Table 9. Primers used for ChIP assays.

PCK1	Primer sequence 5' -> 3'
Region 1: Forward (-477/-457 bp) and Reverse (-312/-292 bp). **PCR product 185 bp**	FW: TGGCTCAGAGCTGAATTTCC<br>RV: GCAGGCTCTTGCCTTAATTG
Region 2: Forward (-280/-260 bp) and Reverse (-145/-125 bp). **PCR product 155 bp**	FW: CAACAGGCAGGGTCAAAGTT<br>RV: GCACGGTTTGGAACTGACTT
Region 3: Forward (-122/-107 bp) and Reverse (-1/+18 bp). **PCR product 140 bp**	FW: CCATGGCTATGATCCAAAGG<br>RV: CAGAGGGAAGGCCAACTGT

p21	Primer sequence 5' -> 3'
Human (Hep3B cells)	FW: GTGGCTCTGATTGGCTTTCTG<br>RV: CTGAAAACAGGCAGCCCAAG
Mouse	FW: GAGACCAGCAGCAAAATCG<br>RV: CAGCCCCACCTCTTCAATTC
