# Supplementary material for: O-GlcNAcylated p53 in the liver modulates hepatic glucose production
Source: Nat Commun. 2021 Aug 20;12:5068. doi: 10.1038/s41467-021-25390-0 (PMC8379189; doi:10.1038/s41467-021-25390-0)
Supplement: Supplementary file 3 — Reporting Summary [file 41467_2021_25390_MOESM3_ESM.pdf]

nature portfolio

Corresponding author(s): Rubén Nogueiras

Last updated by author(s): Jul 19, 2021

Reporting Summary

Nature Portfolio wishes to improve the reproducibility of the work that we publish. This form provides structure for consistency and transparency in reporting. For further information on Nature Portfolio policies, see our Editorial Policies and the Editorial Policy Checklist.

Statistics

For all statistical analyses, confirm that the following items are present in the figure legend, table legend, main text, or Methods section.

n/a	Confirmed	
☐	☒	The exact sample size (n) for each experimental group/condition, given as a discrete number and unit of measurement
☐	☒	A statement on whether measurements were taken from distinct samples or whether the same sample was measured repeatedly
☐	☒	The statistical test(s) used AND whether they are one- or two-sided<br>*Only common tests should be described solely by name; describe more complex techniques in the Methods section.*
☐	☒	A description of all covariates tested
☐	☒	A description of any assumptions or corrections, such as tests of normality and adjustment for multiple comparisons
☐	☒	A full description of the statistical parameters including central tendency (e.g. means) or other basic estimates (e.g. regression coefficient) AND variation (e.g. standard deviation) or associated estimates of uncertainty (e.g. confidence intervals)
☐	☒	For null hypothesis testing, the test statistic (e.g. F, t, r) with confidence intervals, effect sizes, degrees of freedom and P value noted<br>*Give P values as exact values whenever suitable.*
☒	☐	For Bayesian analysis, information on the choice of priors and Markov chain Monte Carlo settings
☒	☐	For hierarchical and complex designs, identification of the appropriate level for tests and full reporting of outcomes
☐	☒	Estimates of effect sizes (e.g. Cohen's d, Pearson's r), indicating how they were calculated

Our web collection on statistics for biologists contains articles on many of the points above.

Software and code

Policy information about availability of computer code

Data collection	Haematoxylin/eosin staining and immunohistochemistry images were obtained using Cell Sense 1.17 software from Olympus.
Data analysis	Microsoft Excel, Image-J software and GraphPad Prism 8.

For manuscripts utilizing custom algorithms or software that are central to the research but not yet described in published literature, software must be made available to editors and reviewers. We strongly encourage code deposition in a community repository (e.g. GitHub). See the Nature Portfolio guidelines for submitting code & software for further information.

Data

Policy information about availability of data

All manuscripts must include a data availability statement. This statement should provide the following information, where applicable:

- Accession codes, unique identifiers, or web links for publicly available datasets
- A description of any restrictions on data availability
- For clinical datasets or third party data, please ensure that the statement adheres to our policy

The authors declare that all data supporting the findings of this study are available within the article in the Supplementary Source Data file. Any further information can be provided upon request.

Field-specific reporting

Please select the one below that is the best fit for your research. If you are not sure, read the appropriate sections before making your selection.

[x] Life sciences   [ ] Behavioural & social sciences   [ ] Ecological, evolutionary & environmental sciences

For a reference copy of the document with all sections, see nature.com/documents/nr-reporting-summary-flat.pdf

Life sciences study design

All studies must disclose on these points even when the disclosure is negative.

Sample size	For animal experiments, sample size was chosen based on similar previous studies of our group and on the basis of literature documentation of similar wellcharacterized experiments. We try to use the fewest number of mice to achieve statistical significance. The sample size and the power to detect the differences between experimental groups are provided in figure legends and in the Statistical Analysis section in Methods. Sample size on human data was only limited by availability.<br><br>Porteiro, B., Fondevila, M., Delgado, T. et al. Hepatic p63 regulates steatosis via IKK!/ER stress. Nat Commun 8, 15111 (2017). https://doi.org/10.1038/ncomms15111<br><br>Folgueira, C., Beiroa, D., Porteiro, B. et al. Hypothalamic dopamine signalling regulates brown fat thermogenesis. Nat Metab 1, 811–829 (2019). https://doi.org/10.1038/s42255-019-0099-7
Data exclusions	Samples of animals and cell cultures were excluded whether their values were outside the 2SD range. Criteria were pre-established. See Statistics section. For patients, the inclusion and exclusion criteria and treatment protocol are mentioned in the Statistical Analysis section. No data from patients have been excluded.
Replication	Animal experiments were performed with sufficient sample sizes as stated above. Every experiment was repeated at least twice and all attempts to replicate experiments were successful.
Randomization	Before start an experiment, all mice groups were made with set of animals of the same sex, age and similar body weight. See Statistical analysis section. The distrubition of the animals with the same genotype to receive the treatments was randomized. For patients, the inclusion and exclusion criteria and treatment protocol are mentioned in the Statistical Analysis section.
Blinding	For practical reasons, the investigators were not blinded to in vivo treatments, since we need to know which animals are injected with each treatment. After collecting the samples, the investigators were blinded to group allocation during data collection and analysis.

Reporting for specific materials, systems and methods

We require information from authors about some types of materials, experimental systems and methods used in many studies. Here, indicate whether each material, system or method listed is relevant to your study. If you are not sure if a list item applies to your research, read the appropriate section before selecting a response.

Materials & experimental systems

n/a	Involved in the study	
[ ]	[x]	Antibodies
[ ]	[x]	Eukaryotic cell lines
[x]	[ ]	Palaeontology and archaeology
[ ]	[x]	Animals and other organisms
[ ]	[x]	Human research participants
[x]	[ ]	Clinical data
[x]	[ ]	Dual use research of concern

Methods

n/a	Involved in the study	
[x]	[ ]	ChIP-seq
[x]	[ ]	Flow cytometry
[x]	[ ]	MRI-based neuroimaging

Antibodies

Antibodies used	The following antibodies were used:<br>p53: cell signaling (2524).<br>Phosphoenolpyruvate Carboxykinase 1 (PCK1): Abcam (ab70358)<br>O-linked N-Acetylglucosamine (O-GlucNAc): Abcam (ab2739)<br>Glyceraldehyde 3- phosphate Dehydrogenase (GAPDH): Merck (CB1001)<br>Phospho-CREB (pCREB) (Ser 133): Cell Signaling (9198)<br>Phospho-AKT (pAKT) (Ser 473): Cell Signaling (9271)

	Phospho-Pyruvate Dehydrogenase E1-alpha subunit antibody (pPDH): Abcam (ab177461)<br>Pyruvate Carboxylase (PC): Abcam (ab128952)<br>Glucose-6-phosphatase: Abcam (ab83690)<br>p21: Cell Signaling (2947T)<br>O-GlcNAc transferase (OGT): Cell Signaling (D1D8Q)<br>All primary antibodies used in this report were obtained from commercial sources.<br>The validation information can be found online for<br>antibodies from Cell Signaling (https://www.cellsignal.com/about-us/cst-antibodyvalidation-principles), Abcam (https://www.abcam.com/primary-antibodies/howwe-validate-our-antibodies) and Santa Cruz (https://www.scbt.com/p/p53-antibody-do-1?productCanUrl=p53-antibody-do-1&_requestid=328194)
Validation	The validation information can be found online for<br>antibodies from Cell Signaling (https://www.cellsignal.com/about-us/cst-antibodyvalidation-principles), Abcam (https://www.abcam.com/primary-antibodies/howwe-validate-our-antibodies) and Santa Cruz (https://www.scbt.com/p/p53-antibody-do-1?productCanUrl=p53-antibody-do-1&_requestid=328194)

Eukaryotic cell lines

Policy information about cell lines

Cell line source(s)	THLE-2 cells (The Global Bioresource Center; CRL-2706) and Hep3B cells (European Collection of Authenticated Cell Cultures, Sigma; #86062703). All cell lines have been authenticated by STR profiling (ATCC).
Authentication	THLE-2 cells express phenotypic characteristics of normal adult liver epithelial cells. They are nontumorigenic when injected into athymic nude mice, have near-diploid karyotypes, and do not express alpha-fetoprotein. THLE-2 cells metabolize benzo [a]pyrene, N-nitrosodimethylamine, and aflatoxin B1 to their ultimate carcinogenic metabolites that adduct DNA, which indicates functional cytochrome P450 pathways. Other enzymes involved in metabolism of chemical carcinogens, such as epoxide hydrolase, NADPH cytochrome P450 reductase, superoxide dismutase, catalase, glutathione S-transferases, and glutathione peroxidase are also retained by THLE cells.<br>Hep3b cells do not express p53 gene.
Mycoplasma contamination	Our lab has routine testing to make sure no mycoplasma contamination in the cell culture system.
Commonly misidentified lines<br>(See ICLAC register)	Our cell are not listed in the database of commonly misidentified cell lines

Animals and other organisms

Policy information about studies involving animals; ARRIVE guidelines recommended for reporting animal research

Laboratory animals	8-weeks-old wild type {WT) mice (C57BL/6), male mice that ca rrying floxed p53 al leles {with a C57BL/6 background) and conditional p53 liver KO mice were housed in air-conditioned rooms {22-24ºC) under a 12:12 h light/dark cycle and controlled conditions of humidity (40%). p53 floxed mice were obtained from The Jackson Laboratory. Mice lacking p53 in the liver were generated in our laboratory crossing p53 floxed mice with Alfp-Cre mice, which express the ere-recombinase open reading frame {ORF) under the control of both the mouse albumin regulatory elements and the alfa-fetoprotein enhancers (AlfpCre transgen e), configuration that mimies the genomic organization of the mouse albumin gene.<br>All experiments and procedures involved in this study were reviewed and approved by the Ethics Committee of the University of Santiago de Compostela, in accordance with European Union normative far the use of experimental animals.<br><br>All this information about animal species, sex, strain, provider, etc are mentioned in the Material and Methods section.
Wild animals	N/A
Field-collected samples	N/A
Ethics oversight	Faculty Animal Committee at the University of Santiago de Compostela

Note that full information on the approval of the study protocol must also be provided in the manuscript.

Human research participants

Policy information about studies involving human research participants

Population characteristics	All the patients: 30 normoglycemic (15 women/15 men) and 30 type 2 diabetes (15 women/ 15 men) with an age average of 50 year-old included in the study were obese, and were further subclassified acording to their normoglyce mia or type 2 diabetes. No self-selection bias impacted results.<br>All patients were recluted from the Endocrinology Department of the University Clinic of Navarra. Clinical studies were approved, from an ethical and sc ientific standpoint, by the Hospital's Ethical Committee and were conducted in accordance with the principies of the Declaration of Helsinki with patients giving their informed consent for participation. The inclusion a nd exclusion criteria and treatment protocol are mentioned in the Material and Methods section.
Recruitment	Inclusion criteria encompassed a complete diagnostic work-up including physical examination, laboratory investigation, ultrasound echography and liver biopsy consistent with the diagnosis of non-alcoholic fatty liver disease (NAFLD) according to the criteria of Kleiner and Brunt by an expert pathologist masked to all the results of the assays.
Ethics oversight	Clínica Universidad de Navarra Ethical Committee

Note that full information on the approval of the study protocol must also be provided in the manuscript.
